# Identifying who lives in a care home—a challenge to be conquered

**DOI:** 10.1093/ageing/afx200

**Published:** 2018-01-16

**Authors:** Jennifer Kirsty Burton, Bruce Guthrie

**Affiliations:** 1 Alzheimer Scotland Dementia Research Centre & Centre for Cognitive Ageing and Cognitive Epidemiology, University of Edinburgh; 2 Population Health Sciences Division, University of Dundee Medical School

Care home residents in the UK outnumber hospital inpatients threefold [1] and yet our knowledge about their needs, care and outcomes is staggeringly poor, not least because there is no central register of care home residents. Identifying who lives in a care home is therefore difficult. Large care data collection systems which have been adopted by care home providers in North American and Europe for service evaluation and research, such as the Inter-RAI (resident assessment instrument) [2] and Minimum Dataset (MDS) [3] are not routinely used in the UK [4]. These data can be extracted and linked to hospital data to evaluate outcomes.

Housley *et al.* describe a novel method to more accurately identify care home residents using only their postal address [5]. The authors used computational matching techniques to match patients’ free-text address data to the Care Quality Commission registered addresses of care homes in the East Midlands region. Their method achieved a higher positive predictive value (100%) than the existing approach used by the Nuffield Trust (87%) which uses postcode matching combined with individual age >75 years, meaning those identified as care home residents are more likely to be care home residents [6].

One of the study’s starkest findings was that in the Trust none of the care home residents admitted were identified as care home residents in the hospital patient administration system, since they were coded as admitted from their ‘usual residence’ [5]. While such coding is true, it means that nursing home residents remain invisible in data used for NHS management and improvement and for research. Inadequate coding like this is common [7]. Although it would clearly be better if routine systems better recorded care home residents, approaches based on address matching provide a pragmatic way of identifying this population for research in the meantime.

While the approach in the paper shows considerable promise, the generalisability of the method proposed is uncertain. The authors have shown excellent predictive performance in the dataset used to derive the rule, but such internal validation does not guarantee good performance in other datasets. The external validation work presented in a second Trust relied on internal Trust procedures to identify care homes, rather than any independent gold-standard comparison. Furthermore, the second Trust had specifically invested in a rigorous approach to identifying care home residents, potentially resulting in more consistent and accurate recording of addresses than will happen in other organisations. As the matching relies on similarities, this may influence the findings and so further external validation across a larger range of organisations is required.

Care home research in the UK has tended to rely on well-conducted studies, recruiting individuals in selected care homes, gathering bespoke data [8–10]. While this approach has commendable rigour, included populations may not be representative of the wider care home population and this approach cannot support real-time use for improvement. While focused, smaller-scale research involving residents, families and staff will always be required, data-driven approaches can help ensure this vulnerable and complex population are included in large-scale research and support more evidence-based health and social care policy in a rapidly changing system of care.

So, why is identifying those who live in care homes so challenging? Primarily because care home residency is not systematically recorded by the NHS or any other public body. In the UK there has been a shift across the care home sector to greater private provision, with multiple providers of care out with the health and local authority sector [11], meaning systematic national data collection is problematic. There are no UK registers of all care home residents, even though such registers would be technically straightforward to create as part of GP registration and change of address.

As a consequence we are driven to address matching methods, where an individual’s address is compared to a list of registered care services. In addition to the issues highlighted by Housley *et al.* [5], our experience is that there is considerable variation in how care home residents’ addresses are recorded (with or without the care home name, with or without a street address, often missing a complete postcode) with matching further complicated by care homes frequently changing service name and registration, without the residents or their NHS recorded address actually changing. Furthermore, increasing use of care homes for short-stay and intermediate care means that care home residency can be transitory. Other potential sources of care home residency status include using GP electronic health record codes for care home residence or data from local authorities. However, these approaches carry their own potential biases around inclusion, contemporaneousness and representativeness. None are systematically collected for all care home residents.

The ideal solution is systematic recording of care home residency status across the four UK jurisdictions, which is contemporaneous and accurate. Recording at general practice registration and change of address notification would be a potential route to achieve this, but would require joined-up information sharing between primary and secondary care data systems. In the meantime, research and evaluation will rely on address matching. This can be supported by more studies like that by Housley *et al.* [5], but with complete gold-standard ascertainment of residency status in both derivation and validation datasets. Researchers should also publish full details of their method to allow widespread external validation. The ‘prevalence’ of care home residents within the sample must be considered, as care home residents are the minority of the older adult and inpatient population, so tools are likely to always perform well with respect to their specificity and negative predictive value with likely trade-offs between increasing sensitivity and lower positive predictive value. Researchers and commissioners need to examine the performance of different tools to select the method most suitable for their purpose.

The World Health Organization ten priorities for a decade of health ageing recognise the need for quality global data on long-term care and effective use of such data in research [12]. Our understanding and support for the vital role care homes have in providing long-term care can be enhanced through improving the quality of the data available to use for needs assessment, service improvement, and research. Current routine data systems are not fit for purpose, perpetuating the marginalisation of this very vulnerable group.

**Key points**

- Care home residents are difficult to identify in routinely collected NHS data.
- Computational address matching can improve identification and is superior to postcode matching.
- Effort should be made to improve precision in coding care home admissions accurately.
- Routine data research has tremendous potential, but accurate identification of the care home population must be a priority.
